# Changes in glycinergic neurotransmission alter mammalian retinal information processing

**DOI:** 10.3389/fnmol.2025.1564870

**Published:** 2025-05-09

**Authors:** Anneka Joachimsthaler, Katharina Hauf, Anja Armbruster, Shiri Zayit-Soudry, Efrat Naaman, Ido Perlman, Rina Leibu, Alina Kurolap, Hagit Baris Feldman, Jan Kremers, Volker Eulenburg

**Affiliations:** ^1^Department of Ophthalmology, University Hospital Erlangen, Erlangen, Germany; ^2^Animal Physiology, Department of Biology, Friedrich-Alexander-University of Erlangen-Nürnberg, Erlangen, Germany; ^3^Department of Biochemistry and Molecular Medicine, Institute of Biochemistry, Friedrich-Alexander University of Erlangen-Nürnberg, Erlangen, Germany; ^4^Department of Anesthesiology and Intensive Care Therapy, University of Leipzig, Leipzig, Germany; ^5^Department of Ophthalmology, Rabin Medical Center, Petah Tikva, Israel; ^6^The Ruth & Bruce Rappaport Faculty of Medicine, Technion - Israel Institute of Technology, Haifa, Israel; ^7^Department of Ophthalmology, Rambam Human Health Care Campus, Haifa, Israel; ^8^The Genetics Institute and Genomic Center, Tel Aviv Sourasky Medical Center, Tel Aviv, Israel; ^9^School of Medicine, Faculty for Medical & Health Sciences, Tel Aviv University, Tel Aviv, Israel; ^10^Translational Anesthesiology and Intensive Care, Medical Faculty University of Augsburg, Augsburg, Germany

**Keywords:** glycine, synaptic inhibition, retina, amacrine cells, ERG, ON-OFF signalling, mouse, human

## Abstract

Glycine, along with GABA, constitutes the major inhibitory neurotransmitter in the central nervous system. In the retina, glycinergic neurotransmission is primarily used by amacrine cells that are involved in the lateral processing of visual stimuli in the inner retina. We have previously shown that the high-affinity glycine transporter 1 (GlyT1), that is commonly used as a reliable marker for glycinergic amacrine cells in the retina, is essential for glycinergic neurotransmission by these cells. Abolishment of retinal GlyT1 expression results in a breakdown of glycinergic neurotransmission by AII amacrine cells, but most likely also by other glycinergic amacrine cell populations. However, the impact of loss of glycinergic neurotransmission on retinal signal processing and visually guided behavior, has not yet been elucidated. In this study, the effects of loss of retinal GlyT1 expression in glycinergic amacrine cells on the optomotor reflex and on the photopic and scotopic electroretinogram (ERG) responses were analyzed. We show that retinal GlyT1-deficient mice have normal optomotor responses to rotating black and white stripes. When stimuli with sawtooth luminance profiles were used, thereby differentially activating ON and OFF pathways, the GlyT1 deficient mice showed facilitated responses to ON preferring stimuli, whereas responses to OFF preferring stimuli were unchanged. These findings were corroborated by ERG recordings that showed undistinguishable responses after flash stimulation but revealed differences in the differential processing of ON and OFF preferring stimuli. To determine if the function of retinal GlyT1 is conserved in humans, we analyzed ERG recordings from a patient diagnosed with GlyT1 encephalopathy. We show that GlyT1 deficiency results in marked ERG changes, characterized by an almost complete loss of the “photopic hill” phenomenon, a hill-like appearance of the relationship between the b-wave amplitude and log light stimulus strength under background illumination conditions, and reductions in the ERG oscillatory potentials in the dark- and light-adapted states. Both findings are consistent with an altered interaction between ON- and OFF pathways in the retina. Taken together our data show that glycinergic neurotransmission in the retina has important functions in retinal ON and OFF processing both in mice and humans.

## 1 Introduction

Glycine acts as an inhibitory neurotransmitter in the central nervous system (CNS), predominantly in the caudal regions, but also the retina. At glycinergic synapses, binding of glycine to postsynaptic glycine receptors (GlyR) results in an increase in chloride conductance and (in most cells of the mature nervous system) triggers a hyperpolarization of the postsynaptic cell (Legendre, 2001), thereby leading to a sign inversion of the electrical signal across chemical synapses. In the caudal regions of the CNS, glycinergic neurotransmission is controlled by two high affinity glycine transporters, GlyT1 and GlyT2 (Eulenburg and Hulsmann, 2022). Loss-of-function studies in mice demonstrated that GlyT2 mediates the reuptake of glycine from the extracellular space into the presynaptic terminal, thus providing substrates for refilling of synaptic vesicles in glycinergic neurons (Gomeza et al., 2003b). In contrast, GlyT1 is responsible for the clearance of glycine from the synaptic cleft, by transporting it into surrounding glial cells (Gomeza et al., 2003a; Eulenburg et al., 2010). In humans, mutations in the GlyT1 gene result in a disease phenotype named GlyT1 encephalopathy (Alfadhel et al., 2016; Kurolap et al., 2016), which resembles most aspects of the phenotype seen in mice and displays many, but not all, facets of nonketotic hyperglycinemia, a rare inborn metabolic disorder associated with mutations in the glycine cleavage system (Applegarth and Toone, 2004). To date, at least ten individuals from six different families carrying different mutations in the GlyT1 gene were identified (Alfadhel et al., 2016; Kurolap et al., 2016; Hauf et al., 2020; Mademont-Soler et al., 2021), all demonstrating impaired transporter activity, thus suggesting GlyT1 dysfunction to be causative for the disease (Hauf et al., 2020). The most frequently reported symptoms include respiratory failure, hypotonia, arthrogryposis and increased nuchal translucency on prenatal ultrasound (Alfallaj and Alfadhel, 2019).

In the retina, glycinergic amacrine cells are known to form glycinergic synapses onto bipolar cells and ganglion cells, thus modulating retinal output (Wässle et al., 2009). Bipolar- and ganglion cells are part of the vertical signaling pathway in the retina whereas the amacrine cells are part of the lateral processing mechanism. Lateral processing in the proximal retina is achieved by a well ordered but complex microcircuitry involving several highly-specialized cell types, including over 60 known types of amacrine cells, releasing different neurotransmitters and showing different connectivity and stratifications (Baden et al., 2016; Yan et al., 2020). From these, 43 have been described as exclusively GABAergic and 13 express glycinergic markers (Yan et al., 2020). Details of the function of the different amacrine cell types and the consequences of their dysfunction are not yet fully understood.

In retinal glycinergic amacrine cells, only GlyT1 is expressed and was suggested to be essential for the maintenance of high intracellular glycine concentrations within the glycinergic neurons and for low glycine concentration in the extracellular space (Pow, 1998; Pow and Hendrickson, 2000). In agreement with this proposal, loss of GlyT1 expression resulted in a breakdown of glycinergic neurotransmission transduced by AII amacrine cells (Eulenburg et al., 2018) and in marked changes in the signal transmission mediated by these cells. The functional consequences of the changed glycinergic neurotransmission on retinal information processing and on vision have yet to be elucidated.

In this study, we investigated the functional effects of retinal GlyT1 deficiency in a mouse model of retina-specific GlyT1 deficiency (Eulenburg et al., 2018) showing complete loss of GlyT1 expression in AII amacrine cells and possibly also in other glycinergic amacrine cell types. In addition, using ERG recording, we studied the retinal function in a human subject diagnosed with GlyT1 encephalopathy due to general functional GlyT1 deficiency. We show significantly altered optomotor reflexes (OMRs) in the GlyT1 deficient mice, demonstrating the importance of GlyT1 for proper retinal function and visual behavior. These effects are expressed by asymmetric changes in the retinal ON- and OFF-pathway as established by electroretinography (ERG). Different alterations in the ON- and OFF-pathways were also found in the ERGs obtained from the GlyT1 encephalopathy patient.

## 2 Materials and methods

### 2.1 Animals

All animal experiments were performed in accordance with the local regulations of the Animal Care and Use Committee and approved by the Regierung of Mittelfranken (Ref. No: 54.2532.1–15/10 and 54–2532.1-25/13). All experiments were performed on adult C57BL/6J mice of the indicated genotype. Animals had access to food and water ad libitum and were maintained at a 12/12 h light–dark cycle. Mice of both sexes were used. No gender-related differences were observed in the experiments performed in this study. Retinal specific inactivation of GlyT1 expression was achieved by mating of mice carrying a floxed GlyT1 allele (GlyT1^fl^) (Eulenburg et al., 2010) with mice expressing the Cre recombinase under the control of a BAC transgenic GlyT2 promoter (GlyT2-Cre) (Ishihara et al., 2010). Genotyping was performed on genomic DNA isolated from ear biopsy material by PCR using the primers 201/301 (GCAACCTGTCTCACCTGTTCAAC and TGTAGGAAGAATACCCCACTGCG) binding to the genomic region upstream of Exon 3 of the mouse GlyT1 gene, flanking the insertion site of the first LoxP site, and Cre001/Cre101 (ATTGCCTACAACACCCTGCTGC and CCACACCATTCTTTCTGACCCG) binding to the coding region of the Cre recombinase. For simplicity mice with the genotype GlyT1^fl/fl^ / GlyT2-Cre were named GlyT1^GlyT2-Cre^ throughout the manuscript. Previous characterization of this mouse line (Eulenburg et al., 2018) has demonstrated that it carries a retina specific inactivation of GlyT1 expression in all AII amacrine cells and additionally some other -not jet identified- glycinergic amacrine cell populations.

### 2.2 Analysis of the optomotor responses in mice

**Figure 1:**
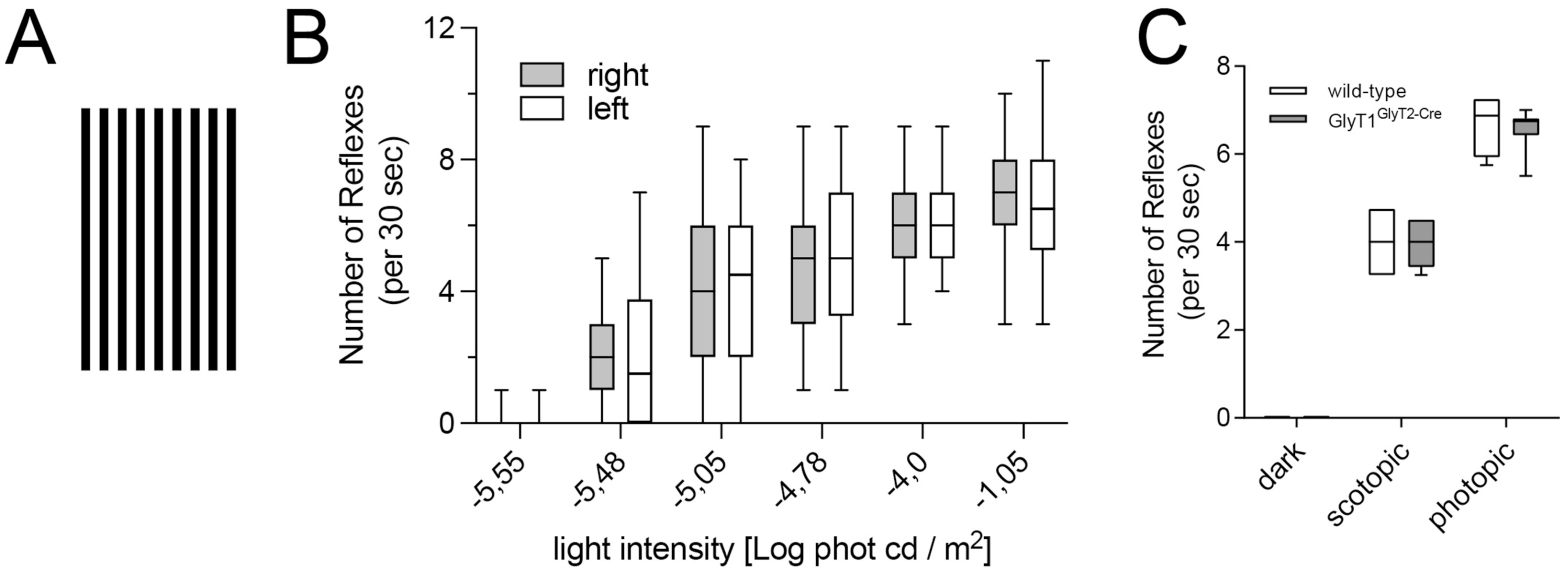
Optomotoric behavior of wild-type and GlyT1^GlyT2-Cre^ mice in response to a rotating black-and-white stripe pattern. **(A)** Square stripe pattern used to elicit optomotoric reflexes (OMRs) in the optokinetic drum. **(B)** Number OMRs elicited in wild-type mice by the bar pattern moving in right or left direction tested under scotopic light conditions as indicated. **(C)** Number of OMRs elicited in wild-type or GlyT1^GlyT2-Cre^ mice under photopic (>400 cd/m^2^) or scotopic conditions (1.6 × 10^−5^ phot cd/m^2^), and in darkness (*n* = 10). No statistically significant differences in OMR numbers between wild-type and GlyT1^GlyT2-Cre^ mice were observed under scotopic or photopic conditions.

**Figure 2:**
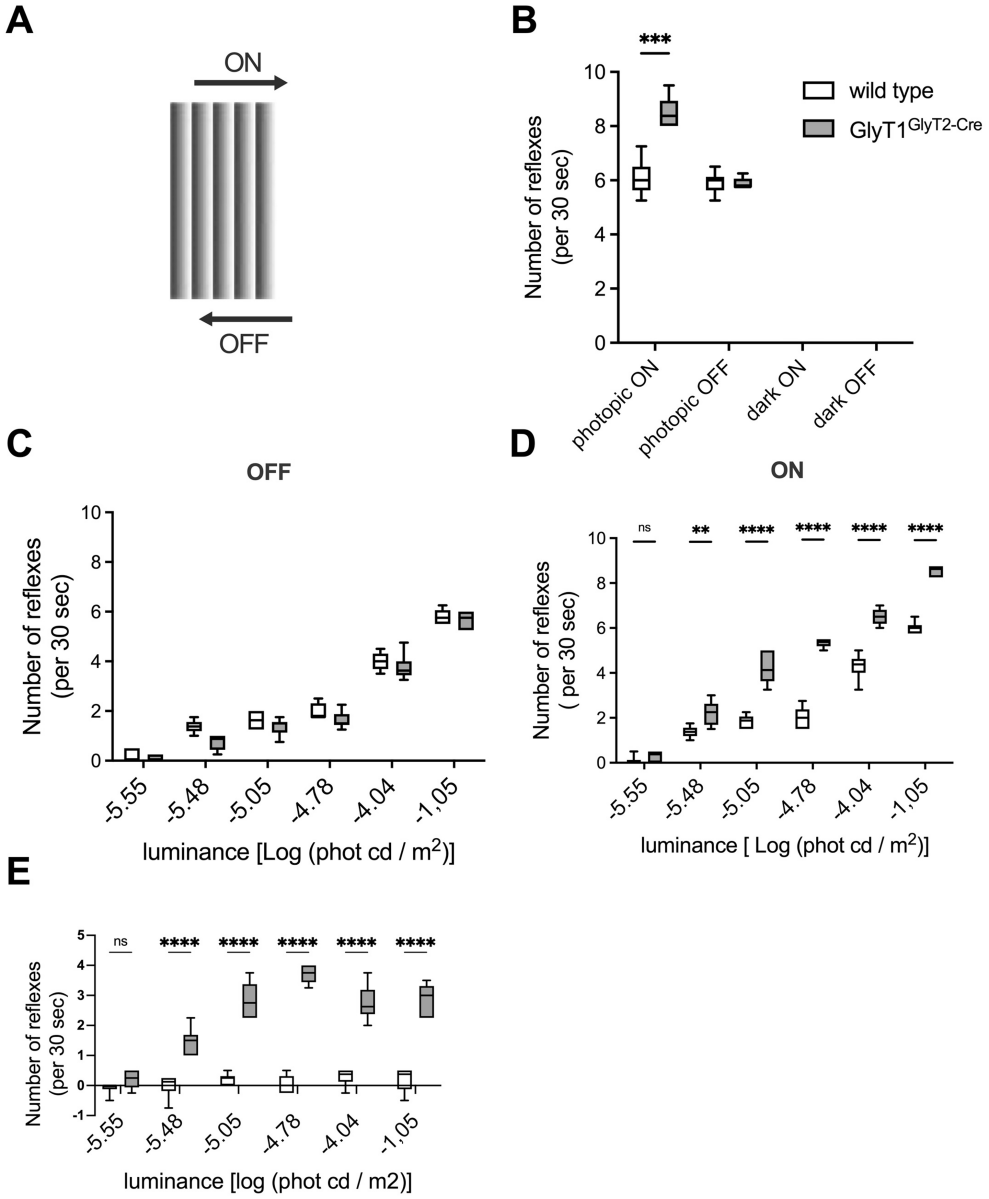
Mouse GlyT1^GlyT2-Cre^ optomotoric behavior with a sawtooth stripe pattern. **(A)** Sawtooth pattern used to elicit OMRs in the optokinetic drum. **(B)** Number of OMRs elicited by the sawtooth pattern tested under photopic conditions (>400 cd/m^2^) or in complete darkness (*n* = 10 per genotype); for ON-stimulation and for OFF-stimulation, the sawtooth pattern was moved clockwise or anticlockwise, respectively. **(C,D)** Number of OMRs elicited under scotopic conditions using the indicated light intensities. OMRs counted for OFF and ON preferring stimuli are presented in **(C,D)** respectively. **(E)** Differences in the OMRs to ON- and OFF-stimuli. Asterisks indicate significant differences between wild-type and GlyT1^GlyT2-Cre^ mice (** *p* < 0.005, **** *p* = 0.0005, student’s t-test).

The optomotor response (OMR) was measured by visual tracking in an optokinetic drum. Adult mice (*n* = 9 per genotype, aged 3–12 month) were used for this study. During experiments, the investigator was blinded about the genotype of the tested mice. Animals were placed on a platform (8.0 cm diameter, 19.5 cm above the bottom of the drum) surrounded by a motorized drum (diameter 29.0 cm, height 55.5 cm), which rotated clockwise or anticlockwise at 3 rpm (18° / sec). The inner wall was lined with images of vertical black and white symmetrically striped grating (4.5 cm wide stripes) or linear gradient (black to white, 4.5 cm wide) stripes (compare Figures 1A, 2A). Contrast between brightest and darkest point of the stripe pattern was, as determined by luminescence measurements > 1:850.

The experiments were performed in a darkened room. Infrared illumination (>800 nm) was used to obtain infrared movies of the mice with an infrared sensitive camera (Sony). For mouse handling under reduced light intensities, the investigator was equipped with a night vision device. The walls of the drums were illuminated by 3 V white light LED, coupled to three light guiding fibers. Light guiding fibers were connected to an opaque diffusor placed under the mouse platform in the optokinetic drum, causing a maximal luminance of the images of 9 × 10^−2^ cd/m^2^ (equals −1.04 log cd/m^2^). The luminance was varied by using neutral density (ND) filters. Photopic measurements were performed at light intensities >400 cd phot/m^2^.

All mice, irrespective of genotype, were maintained on a 12 h light/dark cycle and tested in randomized order in the dark phase, with exception of photopic measurements, which were performed in the light phase. After 20 min adaption to the light conditions, the mouse was placed on the platform and allowed to settle for 20 s. The drum was rotated clockwise for at least 2 min, followed by a stop of the drum for 30 s, and then rotated anticlockwise for 2 min.

The number of optometric reflexes was determined by counting the head tracking movements in 4 × 30 s intervals of the movies. Periods of grooming were excluded from the evaluation.

### 2.3 Mouse electroretinogram (ERG)

Full details of the setup have been described previously (Regus-Leidig et al., 2014; Joachimsthaler and Kremers, 2019). Briefly, animals were dark-adapted overnight prior to ERG measurements. Dim red illumination was used during animal preparation to maintain dark adaptation. Animals were anesthetized by an intramuscular (i.m.) injection of 50 mg/kg ketamine (Ketavet®, Pfizer) and 10 mg/kg xylazine (Rompun® 2%, Bayer). A subcutaneous (s.c.) injection of saline (300 μL, 0.9%) was delivered to avoid dehydration during anesthesia. The pupils were dilated by a drop of Tropicamide (Mydriaticum Stulln®, 5 mg/mL, Pharma Stulln GmbH) and phenylephrine-hydrochloride (Neosynephrin POS® 5%, Ursapharm). The animal was then placed on a heated platform inside a Ganzfeld stimulator (Roland Consult Q450 SC). Needle electrodes were placed s.c. at the tail base and on the forehead between the eyes, serving, respectively, as ground and reference electrodes. Contact lens electrodes (Ø3.2 mm; Mayo Corporation) filled with Corneregel® (Dr. Mann Pharma) served as active electrodes. The mice were allowed 10 min of dark adaptation. Recordings (RetiPort system; Roland Consult) included the scotopic threshold response (STR; white LED flashes, −4.5, −4.4, and −4.3 log phot cd.s/m^2^, 60–80 averages; white LEDs: 1 phot cd.s/m^2^ = 1.624 scot cd.s/m^2^), to assay rod-driven retinal ganglion cell physiology (Saszik et al., 2002) followed by mesopic rapid-ON/OFF 4 Hz sawtooth paradigms (red LEDs, 0 log phot cd/m^2^ after 5 min adaptation, 80 s measurements; red LEDs: 1 phot cd/m^2^ = 0.04 scot cd/m^2^) to assess ON- and OFF-retinal activity separately (Kremers et al., 2014) Lastly, a scotopic flash ERG protocol was recorded (2 min of adaptation, −0.7, 0.3, 0.8 log phot cd.s/m^2^ white LED flashes, 4–8 averages) to examine outer retinal function (Frishman, 2006). The total duration of scotopic ERG measurements was approx. 45 min. Photopic measurements were done on a second cohort of mice after 5 min adaptation to white light background (25 phot cd/m^2^ equals 1.4 log phot cd/m^2^). Photopic recordings included photopic white light flashes, −0.2, 0.3, 0.8, 1.0 and 1.2 log phot cd.s/m^2^, 20 averages, to examine the integrity of cone-driven responses in the outer retina and ganglion cells (Frishman, 2006), and a photopic rapid-ON/OFF 4 Hz sawtooth paradigm (60 phot cd/m^2^–1.8 log phot cd/m^2^ - white light, after 5 min adaptation, 40 s measurement) to investigate a possible effect of the GlyT1 knockout on cone-driven ON- and OFF-responses. The total duration of the photopic measurements was approximately 20 min.

**Figure 3:**
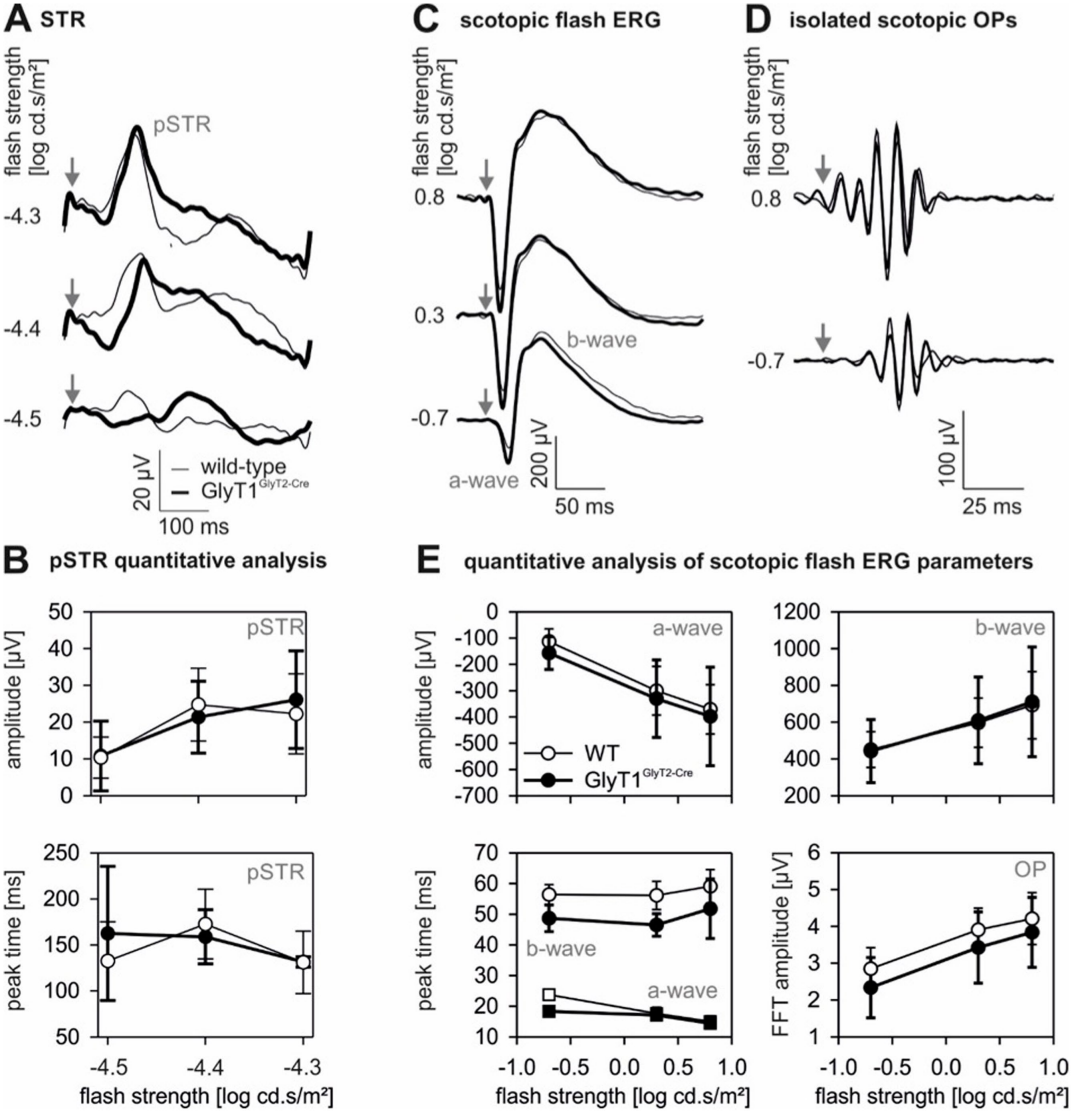
Scotopic flash-ERGs. Summary of scotopic threshold responses **(A)**, and of scotopic flash ERGs (after separation of the OPs obtained from the inverse Fourier transform of the low frequency portion of the original signal) for different flash strengths **(C)**. The responses shown in **(D)** show the isolated OPs (obtained from the inverse Fourier transform of the high frequency portion of the original signal). The arrows indicate time of flash. The thin line represents mean responses of wild-type animals (*n* = 8) and the thick line represents the mean responses measured in GlyT1^GlyT2-Cre^ mice (*n* = 10). The graphs in **(B)** depict the amplitudes (upper graph) and peak times (lower graph) of the pSTR. The graphs in **(E)** show the amplitudes of a-wave (upper left plot), b-wave (upper right plot) and the OPs (lower right plot). The peak times of the a- and b-wave are displayed in the lower left plots. Open symbols represent wild-type data; filled symbols represent GlyT1^GlyT2-Cre^ data. Data points are shown as mean ± sd.

**Figure 4:**
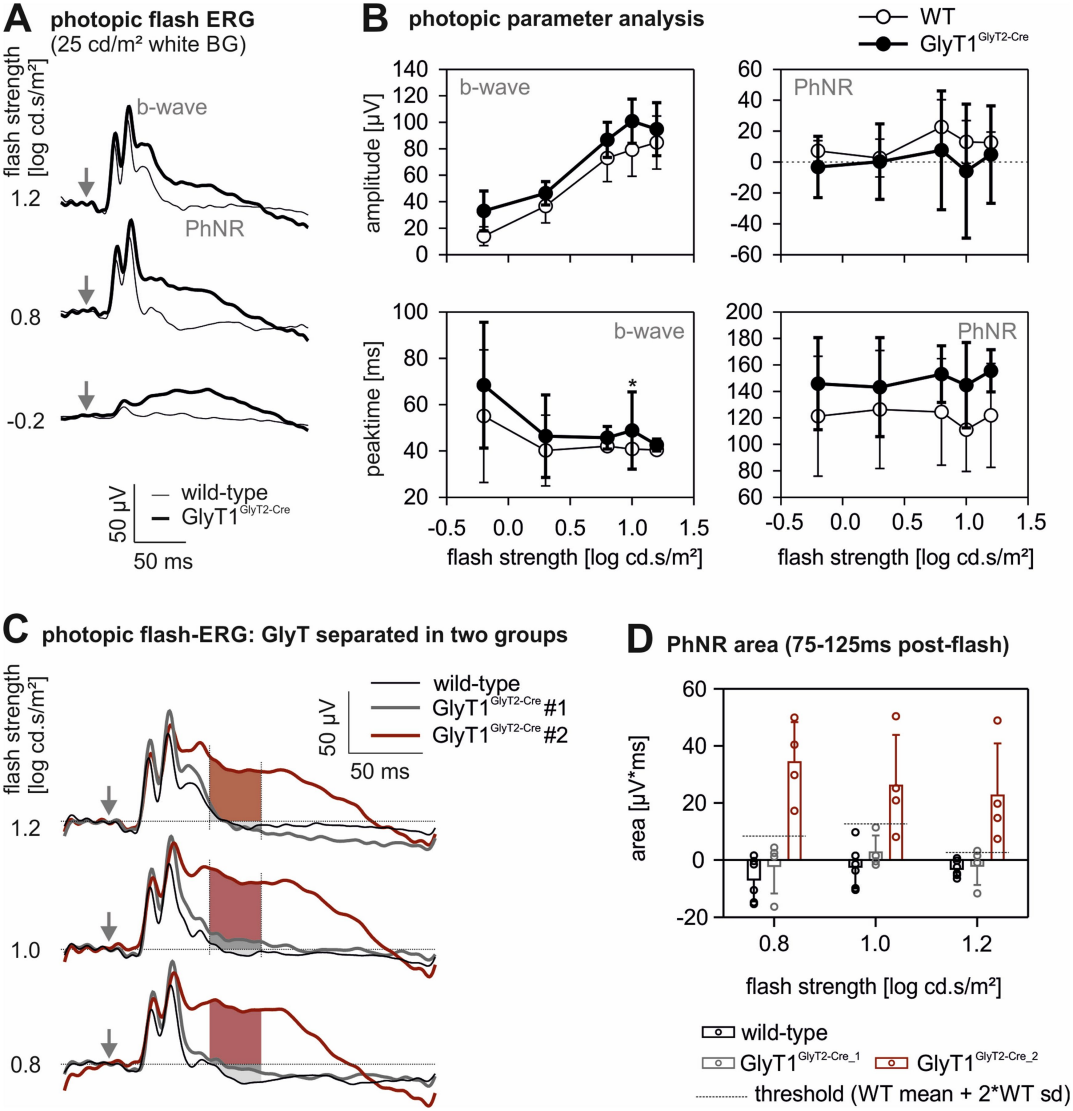
Photopic flash ERGs. Summary of the photopic flash ERG **(A)** at −0.2, 0.8 and 1.2 log cd.s/m^2^ flash strength (flash stimulation on top of a 25 cd/m^2^ (equals 1.4 log cd/m^2^) white background). Arrows indicate time of flash. The thin line represents mean responses in the wild-type animals (*n* = 6) and the thick line represents mean responses in GlyT1^GlyT2-Cre^ mice (*n* = 8). **(B)** Amplitudes (upper plots) and peak times (lower plots) of the b-wave and PhNR as a function of flash strength. Open symbols represent wild-type data; closed symbols represent GlyT1^GlyT2-Cre^ data. Data is shown as mean ± sd. Asterisk represents significant difference between the two genotypes (*: *p* < 0.05). **(C)** Mean photopic flash ERGs as in **(A)**; however, the GlyT1^GlyT2-Cre^ data are subdivided into two groups (of four animals each) dependent upon their PhNR responses. The responses for the three strongest flashes are shown. The shaded area depicts the time window after flash where the areas under the response curves (AUC) are calculated. **(D)** Calculated AUCs for PhNRs, as displayed in **(C)**, for wild-type (black), GlyT1^GlyT2-Cre^ #1 (grey) and GlyT1^GlyT2-Cre^ #2 (red) data, and for the three strongest flashes. Histograms and error bars depict mean ± sd. Symbols indicate individual data. The grey horizontal line segments indicate the thresholds for subdividing GlyT1^GlyT2-Cre^ animals as described in the text.

ERG responses were amplified 100,000 times, band-pass filtered (1–300 Hz) and digitized with a sampling frequency of either 2,048 Hz for flash ERGs or 1,024 Hz for sawtooth and sine flicker ERGs (RetiPort system; Roland Consult, Brandenburg, Germany). All data were analyzed with custom written Matlab (The Math Works) programs. To isolate the oscillatory potentials from a- and b-wave in flash ERG responses, a variable filter was used on the Fourier transform of the response. In the frequency domain, the amplitude plot showed two regions that were separated by a minimum at about 60 Hz. The low frequency portion was attributed to the a- and b-wave and PhNR, whereas the high frequency portion was exclusively attributed to the faster oscillatory potentials [OPs; (Harazny et al., 2009)] We defined the amplitude of the OPs as the maximal amplitude of the high frequency portion between 60 and 100 Hz. The amplitudes and latencies of the a- and b-wave and the photopic negative response (PhNR) of flash ERGs were obtained after inverse Fourier transform of the low frequency portion. The components were defined as follows (illustrated in Figures 3, 4): amplitude of the a-wave was measured from baseline (the first five data points of recording) to the trough of the a-wave. The amplitude of the b-wave was defined as the amplitude between the trough of the a-wave to the peak of the b-wave. Latencies were calculated from time-of-flash to trough or peak. As the photopic a-wave is small in mice (< 10 μV), it was disregarded. For the photopic flash ERG, the amplitude of the PhNR was measured from baseline to its trough. Since the PhNR is a very slow wave component, it can be contaminated by slow potentials caused by breathing activity. To minimize this effect, we calculated the area under the response curve in a time window between 75 and 125 ms post-flash (denoted as PhNR area). The responses of the two eyes of each animal were averaged, if the difference of the maximum b-wave amplitude (at 0.8 log phot cd.s/m^2^ flash for scotopic conditions and at 1.2 log phot cd.s/m^2^ for photopic conditions) between the two eyes was smaller than 10%. If the difference was larger than 10%, only the eye with the larger amplitude was included in the analysis.

### 2.4 Clinical patient assessment

The patient was previously reported as patient 1 in Kurolap et al. (2016). The 2.5-year-old girl underwent a bedside ophthalmological evaluation, including assessment of the occurrence of nystagmus and examination of the anterior segment. Pharmacological pupil dilation with topical tropicamide facilitated an ophthalmoscopic examination of both eyes.

To evaluate the functional status of the retina, full-field electroretinogram (ff-ERG) recording was performed according to the ISCEV guidelines at the time of investigation (McCulloch et al., 2015). The ERG responses were simultaneously recorded from both eyes (UTAS BigShot, LKC Technologies, Gaithersburg, Maryland, USA) after pupil dilation and topical corneal analgesia. DTL corneal electrodes were used as active electrodes and cup EEG electrodes, placed on the temporal canthus of each eye as reference electrode, and a common ground electrode was placed on the forehead between the two eyes. The light-adapted ERG was recorded under white background illumination of 30 cd/m^2^, using white light stimuli of increasing strengths covering a range of approximately 3.3 log units (0.1 to 200 cd.s/m^2^) in addition to a 30-Hz flicker (2.5 cd.s/m^2^). Following 20 min of dark adaptation, a series of white light stimuli of increasing strength covering a strength range of 4.6 log units (0.005 to 200 cd.s/m^2^) were employed. Each ERG response was an average of three consecutive identical stimuli, having a variable interstimulus interval depending upon state of adaptation and strength of flash.

ERG analysis was based on amplitude and implicit time measurements, determined as the measurement that were used for the mice: The a-wave amplitude was measured from the baseline to the trough of the negative wave, and the b-wave amplitude was measured from the trough of the a-wave to the peak of the b-wave. The b-wave implicit time was measured from stimulus onset to the peak of the positive b-wave. The OPs were obtained using a pre-defined protocol of the LKC system. The sum of the OPs was computed from the trough of the a-wave to the peak of the b-wave in order to include only OPS on the ascending part of the b-wave. ERG responses were compared to those from healthy adult volunteers, since no age matched control recordings were available.

### 2.5 Statistics

For statistical analyses, all data were tested for Gaussian distribution with a Shapiro–Wilk-test. If Gaussian-distribution could be assumed, a two-sided, unpaired t-test was performed to check for significant differences between genotypes. If the Shapiro–Wilk-test did not suggest a Gaussian-distribution, a non-parametric Mann–Whitney-U-test was performed to compare genotypes. For the flash-ERGs, the results were corrected after Bonferroni for multiple testing. Bonferroni-correction factors are given in the results. There was no statistical evaluation on the ERG data performed, since only one patient was available for analysis and an age matched ERGs from control groups were not available.

## 3 Results

### 3.1 Retinal GlyT1 deficiency in mice results in marked changes in optomotor responses

To determine possible behavioral consequences of the changed retinal processing in mice carrying a retina specific GlyT1 deficiency (GlyT1^GlyT2-Cre^ mice), we analyzed the optomotor reflex (OMR) elicited by a black and white stripe pattern (Figure 1A) in an optokinetic drum under different illumination conditions. As a readout, we used the frequency of head-movement, caused by the optokinetic nystagmus elicited by a rotating drum surrounding the animal (Tauber and Atkin, 1968). In complete darkness or under very low luminance conditions, wild-type mice did not show any head movement associated with the rotating black and white stripe pattern (Figures 1B,C). Robust OMRs were observed under scotopic luminance between −5.5 and −4 log (cd/m^2^) (Figure 1B). The OMR frequency was dependent on the mean luminance, but independent of the direction of rotation (Figure 1B). When these experiments were repeated with GlyT1^GlyT2-cre^ mice, they showed reflexes that were indistinguishable from those of wild-type animals. Similar results were obtained at luminance levels that favor photopic signaling pathways in the retina (> 400 cd phot/m^2^; Figure 1C). In summary, our results demonstrated that GlyT1^GlyT2-Cre^ mice did not show altered visual evoked behavior, compared to wild-type mice, in response to the rotating black and white stripe pattern under a wide range of illumination conditions, from darkness to bright photopic conditions.

To specifically target changes in ON- and/or OFF-specific pathways, we used gratings with sawtooth spatial luminance profiles (Figure 2A). These stimuli may differentially activate ON- and OFF-signal-induced reflexes, depending on the direction of the rotation.

Again, under complete darkness, no OMRs were observed independent of the direction of rotation. Wild-type mice showed similar frequency of OMRs regardless of the direction of rotation under photopic conditions (Figure 2B; *p* > 0.5), revealing no measurable differences between ON- and OFF-mediated responses. When these experiments were repeated with GlyT1^GlyT2-Cre^ mice, however, a significantly increased number of OMRs in response to a photopic stimulus preferring activation of the ON-channels (rapid-ON) were observed as compared to those of wild-type animals (Figure 2B; *p* ≤ 0.005). In contrast, the OMRs to stimuli preferring OFF signaling in the retina (rapid-OFF stimuli) were like those of the wild-type mice (Figure 2B; *p* ≥ 0.5). To determine if this difference is specific for photopic conditions or if it is also present under scotopic conditions, the experiment was repeated under scotopic luminance conditions. At −5.5 Log (cd /m^2^), all mice showed almost no reactions. At all other luminance, wild-type mice showed similar reaction frequencies to rapid-ON and -OFF stimuli. In contrast, GlyT1^Glyt2-cre^ mice displayed significantly more OMRs to rapid-ON than to rapid-OFF stimuli (Figures 2C–E; *p* ≤ 0.005) as it was also seen under photopic luminance. When the stripe pattern was inverted GlyT1^Glyt2-Cre^ mice still responded more frequently to the rapid-ON signal, whereas responses to rapid OFF signals were similar to that of wild-type animals (*n* = 3, data not shown) Taken together, these data demonstrate that the changes in retinal processing of visual information caused by the impaired glycine-dependent neurotransmission in GlyT1^GlyT2-Cre^ mice, is not restricted to scotopic conditions but can also be observed under photopic conditions.

### 3.2 Retina specific GlyT1 deficiency results in small and heterogenous changes in the mouse ERG

To determine if the observed OMR differences between transgenic mice and wild-type mice have a retinal equivalent, we compared full-field ERG responses of GlyT1 deficient mice to those of wild type mice. The scotopic threshold response (STR) and the scotopic flash ERG (after removal of the oscillatory potentials – OPs) did not differ significantly between wild-type and GlyT1^GlyT2-Cre^ mice (Figures 3A,C; for quantification see, B, E; *p* ≥ 0.5). Also, the amplitudes of the isolated oscillatory potentials (OPs) were similar in wild-type and GlyT1^GlyT2-Cre^ mice (Figure 3D; *p* ≥ 0.5).

In the photopic flash ERG, however, significant differences between the two mouse genotypes were observed (Figure 4A). Here, the photopic b-wave amplitude was slightly, but not significantly increased in GlyT1^GlyT2-Cre^ mice (Figure 4B upper left plot; *p* > 0.05). The b-wave implicit time was slightly delayed in GlyT1^GlyT2-Cre^ mice and reached significance only for 1 log cd.s/m^2^ flashes (Figure 4B lower left plot; Kruskal-Wallis test: *p* ≤ 0.005 after Bonferroni correction for multiple testing). The averaged photopic flash-ERG waveforms between about 60 and 180 ms post stimulus was more positive in the GlyT1^GlyT2-Cre^ mice when compared with those of wild-type mice. Based on these findings, we assumed that the amplitude of the photopic negative response (PhNR) – a slow component which represents ganglion cell activity (Frishman, 2006) – might be significantly altered in GlyT1^GlyT2-Cre^ mice. We, therefore, determined the minimum within this time window and calculated the potential difference relative to baseline (positive values indicating that the potential was below baseline). Owing to the large inter-individual differences, the ERG responses did not differ significantly between the two groups (Figure 4B upper right plot). Reassessment of the data revealed that the GlyT1^GlyT2-Cre^ mice could be sub-classified into two groups: one in which the late potential was similar to that of the wild-type mice (*n* = 4; here called GlyT1^GlyT2-Cre^ #1), and a second group with altered late potentials (*n* = 4; called GlyT1^GlyT2-Cre^ #2). To classify the GlyT1^GlyT2-Cre^ mice we defined a threshold using the wild type data. If the PhNR amplitude of a GlyT1^GlyT2-Cre^ mouse was above our criterion (defined as WT mean + 2*WT standard deviation) the animal was assigned to the GlyT1^GlyT2-Cre^#2 group. The average ERGs of these groups are shown with those of wild-type mice for the three strongest flashes in Figure 4C. We quantified the PhNR amplitude as the area under the ERG traces between 75 and 125 ms post-stimulus (shown as shaded areas in Figure 4C). The amplitudes for the three groups are given in Figure 4D. A statistical analysis of these data is not useful because the division between the two GlyT1^GlyT2-Cre^ groups was based on the late potentials themselves and, therefore, these data should be regarded as an empirical observation. We did not find any common distinction between the two subgroups other than the PhNR. There was no indication that sex or age had an effect on the trace of the PhNR. All animals were recorded by the same researcher with the same equipment.

**Figure 5:**
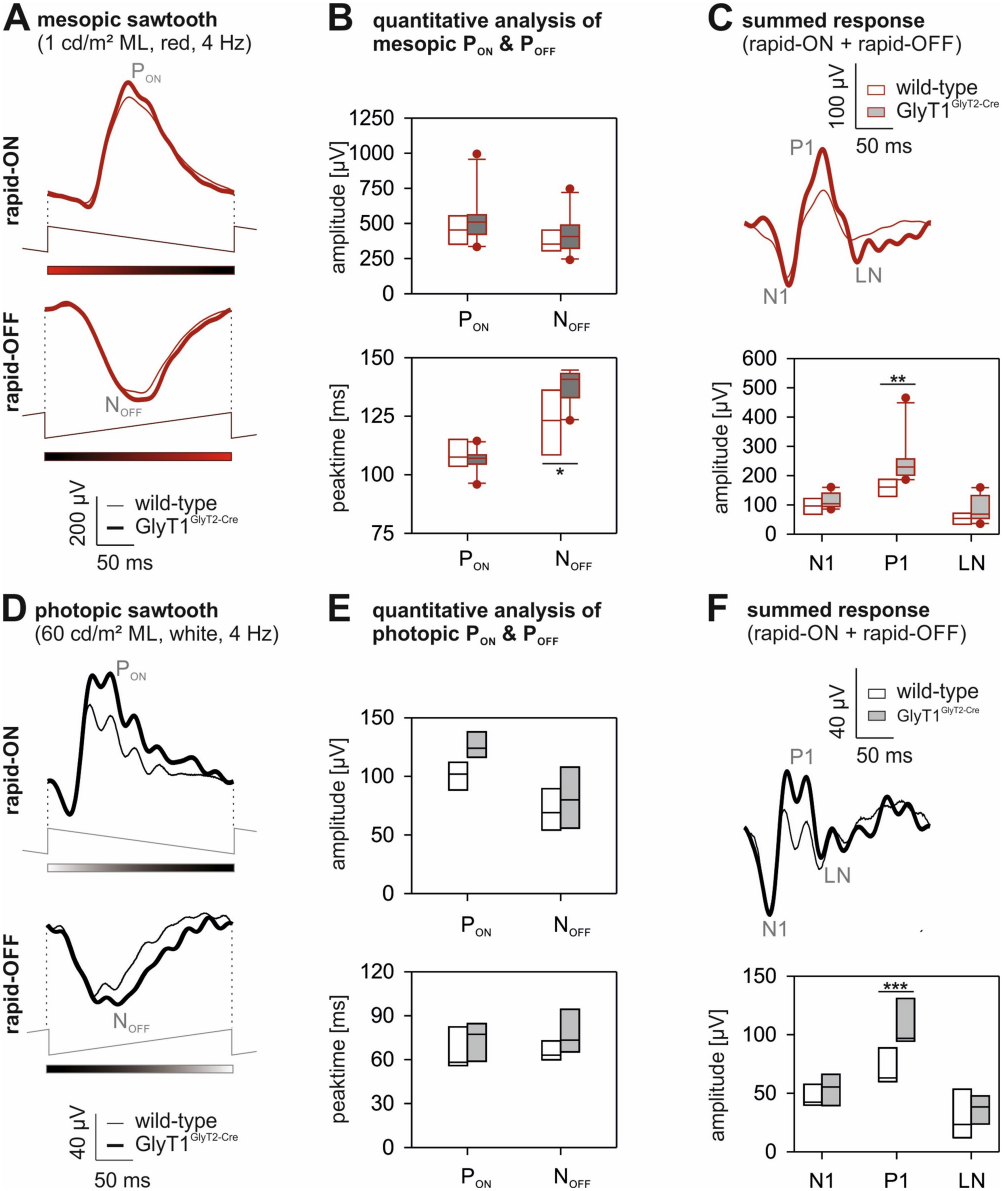
Mesopic and photopic sawtooth-ERGs. Original responses to mesopic **(A)** and photopic **(D)** sawtooth stimuli. The upper plots show responses to rapid-on sawtooth stimuli; the lower plots display responses to rapid-off sawtooth stimuli. The thin line represents mean responses in the wild-type animals (mesopic *n* = 8; photopic *n* = 5) and the thick line represents mean responses in the GlyT1^GlyT2-Cre^ animals (mesopic *n* = 10; photopic *n* = 6). **(B,E)** show the amplitudes (upper plots) and peak times (lower plots) for P_ON_ and N_OFF_ components in mesopic and photopic conditions, respectively. Box plots show median and interquartile range (IQR). Whiskers indicate 1.5 times IQR. Symbols represent outliers. Open boxes: wild-type data; shaded boxes: GlyT1^GlyT2-Cre^ data. **(C,F)** show the summation of ON- and OFF-responses for mesopic and photopic conditions, respectively, same format as in **(A,D)**. The graphs show box plots for N1, P1 and LN component amplitudes. Same format as in **(B,E)**. Asterisks represent a significant difference between the two genotypes (^*^ *p* < 0.05, ^**^ *p* < 0.005 and ^***^ *p* < 0.001).

To separate responses to luminance increments (ON-) and decrements (OFF-), two stimulus types with sawtooth temporal profiles were used (Lee et al., 1993; Pangeni et al., 2012; Kremers et al., 2014); a rapid-ON ramp-OFF and a rapid-OFF ramp-ON to elicit mainly ON- and OFF-responses, respectively. The responses were measured under mesopic (1 cd/m^2^) and photopic (60 cd/m^2^) conditions (Figures 5A,D respectively). In both mesopic and photopic conditions, the amplitudes of the ON- and OFF-responses were slightly increased in the GlyT1^GlyT2-Cre^ compared to those of the wild-type mice (Figure 5B upper plot for mesopic conditions; Figure 5E upper plot for photopic conditions). Moreover, the latency of the minimum in the OFF-response (N_OFF_) was significantly increased in the GlyT1^GlyT2-Cre^ mice at mesopic conditions (*p* = 0.034, Mann–Whitney-U test). This asymmetry between ON- and OFF-responses is more obvious when the responses are summed (Pangeni et al., 2012). This summation shows three components, marked as N1, P1 and LN (traces in Figures 5C,F for mesopic and photopic conditions, respectively). The ON–OFF asymmetries were increased in GlyT1^GlyT2-Cre^ mice; this increase was significant for the P1 component both in mesopic (*p* = 0.004, Mann–Whitney-U-test) and in photopic (*p* = 0.029, Mann–Whitney-U test) conditions. To exclude that the observed effect was solely caused by the outliers, a reanalysis of the data was performed after exclusion of these datapoints, which confirmed the significant difference of P1 between genotypes.

### 3.3 Absence of the “photopic hill” in flash ERGs measured in a patient with GlyT1 deficiency

**Figure 6:**
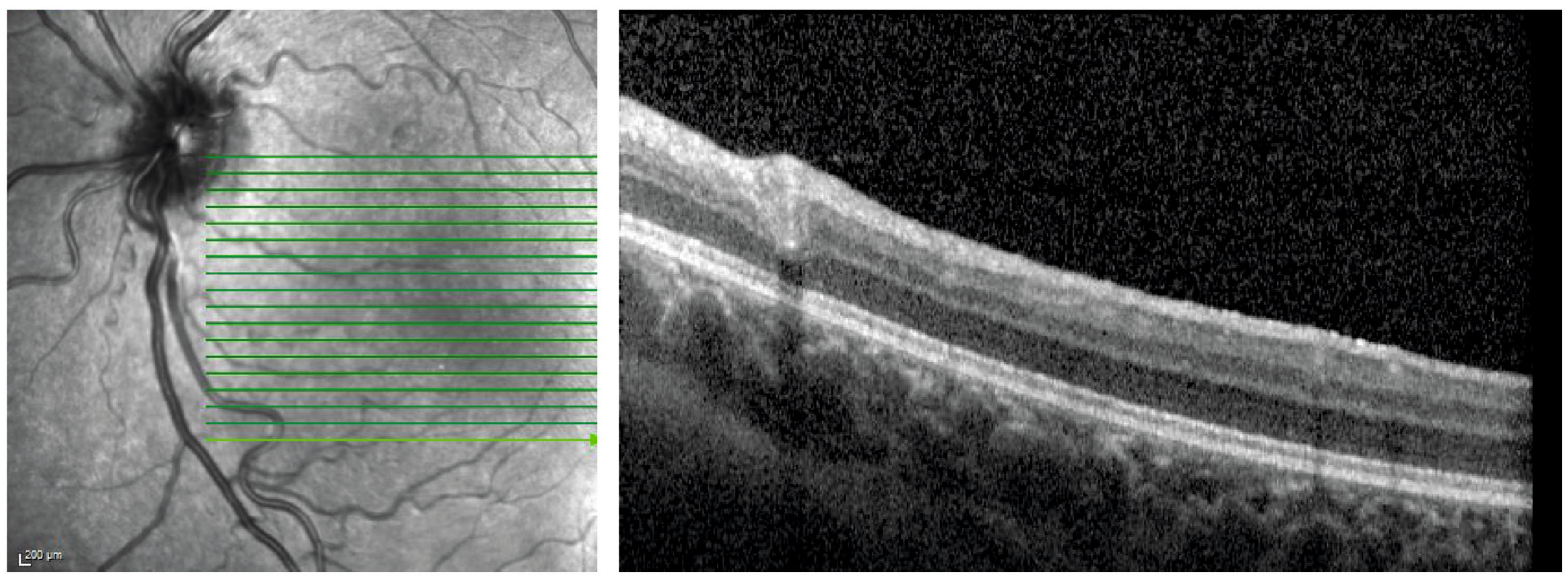
Ophthalmic assessment of a GlyT1 deficient 2.5-year-old patient. Averaged image of OCT sections of the right eye fundus.

To determine if similar ERG abnormalities were observed in humans, the ophthalmological characteristics of a 2.5-year-old patient with GlyT1 encephalopathy caused by mutations resulting in a complete loss of GlyT1 function (Kurolap et al., 2016; Hauf et al., 2020) were assessed. Ophthalmoscopic examination, performed in a bedside manner, revealed clear media in both eyes. The optic nerve head, retinal blood vessels and the macular area were bilaterally unremarkable, and the retina was attached with no apparent morphological abnormalities in each eye. Spectral- domain Optical coherence tomography (SD-OCT) scans revealed normal retinal structure and lamellar architecture (Figure 6).

The flash visual evoked potentials (VEP), recorded with EEG electrodes at the position of the primary visual cortex, consisted of waves of normal amplitudes appearing at normal implicit times (data not shown), indicating normal conductance in the visual pathways from each eye to the primary visual cortex.

**Figure 7:**
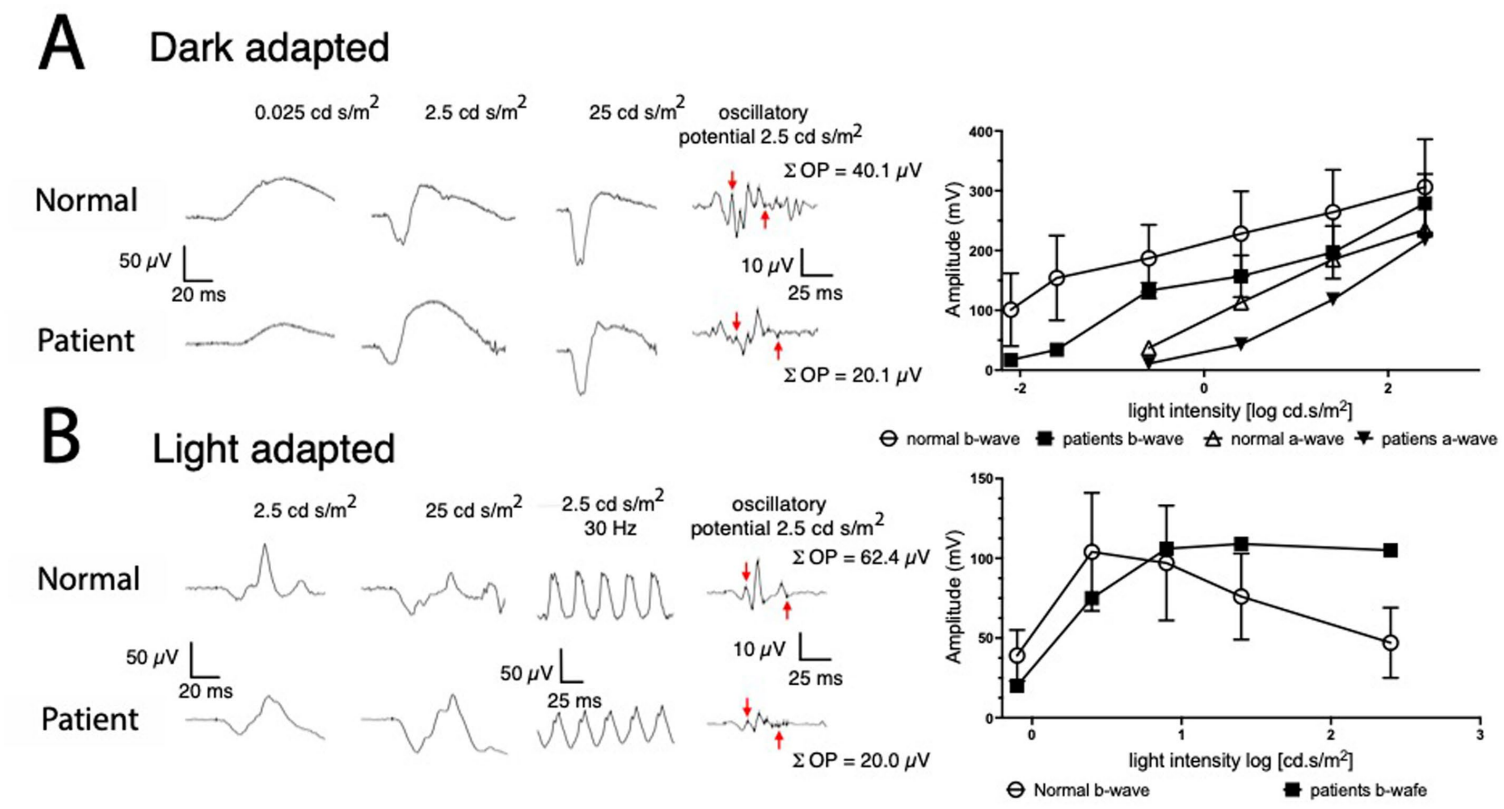
ERG responses of *GlyT*1-deficient patient compared to corresponding responses from a healthy subject. Dark-adapted and light-adapted (background of 30 cd/m2) ERG responses (left parts of **A,B** respectively), are compared between a volunteer with no visual impairment and the *GlyT1*-deficient patient (upper and lower rows of responses respectively). The energy of each stimulus is marked above each column of responses in units of cd*s/m^2^. The rightmost column shows isolated oscillatory potentials (OP) from the responses to the respective 2.5 stimulus. Response–stimulus energy relationships for the dark-adapted a-wave and b-wave and for the light-adapted b-wave showing the mean +/− s.d. of the normal range (*N* = 15) and ERG responses of the *GlyT1*-deficient patient.

To quantitatively assess retinal function, the child underwent full field-ERG recording under dark-adapted and light-adapted conditions and compared to control recordings performed on adult healthy volunteers. The scotopic responses recorded in the dark-adapted state demonstrated mild reduction of the ERG a-wave and b-wave amplitudes, indicating slightly subnormal rod system function in the peripheral retina (Figure 7, left-upper part). The a-wave of the ERG responses that were recorded under light-adapted conditions, are of slightly subnormal amplitudes in response to all light stimuli of all strengths (Figure 7, left lower part). The findings of slightly subnormal amplitudes of ERG a-wave and b-wave under light- and dark-adapted conditions are consistent with previous reports on age-related development of the human ERG (Fulton et al., 2005). However, the light-adapted amplitude-Log I relationship for the light-adapted b-wave showed a monotonic increase in amplitude with increasing stimulus strength, reaching a stable plateau at high stimulus strengths (Figure 7B, right lower part curve with solid squares). This observation contrasts with the “photopic hill “phenomenon that is readily seen when plotting the light-adapted b-wave amplitude as a function of log stimulus strength in subjects with normal retinal function (Wali and Leguire, 1992; Rufiange et al., 2002) as illustrated in Figure 7B (open circles in right lower part) showing the mean +/− s.d. of 20 adult volunteers with normal visual function. Also, the summed amplitudes of the oscillatory potentials, appearing on the ascending part of the ERG b-wave are smaller in the dark- and light-adapted states in the patient compared to those in an adult volunteer with normal vision (numbers are shown within the figure). The amplitudes of the oscillatory potential were summed between the downward arrow and the upward arrow marking the trough of the a-wave and the peak of the b-wave, respectively. These findings are consistent with similar observations in animal studies (Wachtmeister, 1980; Matthews et al., 1989).

## 4 Discussion

In this study, we have investigated the functional and behavioral consequences of retinal GlyT1 deficiency, both in a genetically modified animal model, and in a patient suffering from GlyT1 encephalopathy. We demonstrated that wild-type mice and GlyT1GlyT2-Cre mice showed indistinguishable behavior in response to rotating black and white bars, confirming that the overall processing of visual information is still functional in GlyT1^GlyT2-Cre^ mice. When using stimuli that may differentially stimulate retinal ON- and OFF- pathways, GlyT1^GlyT2-Cre^ mice showed significantly increased oculomotor reflexes (OMRs) in reaction to ON-stimuli compared to wild-type littermates, suggesting an overall stronger retinal signal. Indeed, previous studies have shown that an inhibition of glycine in the retina by strychnine can result in a facilitation of the electrical activity of ON direction sensitive ganglion cells (Sivyer et al., 2019). Taken together these findings are consistent with our previous studies on these mice showing that retinal GlyT1 deficiency results in a breakdown of glycinergic inhibition at least in major populations of glycinergic amacrine cells (Eulenburg et al., 2018). A possible mechanistic explanation for this effect could be changes in the activity of starburst amacrine cells, that have been shown previously to be involved in the modulation of the activity of direction sensitive ganglion cells (Yoshida et al., 2001). Starburst amacrine cells have been shown to use acetylcholine and/or GABA but not glycine as a neurotransmitter (Sethuramanujam et al., 2016), thus a direct influence of GlyT1 deficiency on this cell type is at least unlikely. Although the majority of the inhibitory input onto starburst amacrine cells has been ascribed to be GABAergic input, some glycinergic input has also been reported (Majumdar et al., 2009). Indeed, this glycinergic input has been shown to be involved in direction sensitive ON activity of this cell type and this effect was shown to be mediated predominantly by the glycine receptor subunit α4 (Jain et al., 2022). On ganglion cell level, additional direct glycinergic input onto the ON direction sensitive ganglion cells from VGlut3 positive cells has been shown that most likely use both glutamate and glycine as transmitters (Mani et al., 2023). Here, the glycine dependency might additionally influenced by the stimulus frequency, where higher velocities show stronger glycinergic components (Summers and Feller, 2022). Further investigation is required to establish a firm causal relationship between alterations in glycine dependent signal transmission and the described behavioral phenotype.

In contrast to the OMRs in response to ON preferring stimuli the number of OMRs to OFF-preferring stimuli were similar in both groups independent of the level of illumination, suggesting that the retinal derived signal eliciting this behavioral response is not changed. This contrasts with our previous study which demonstrated that in GlyT1^GlyT2-Cre^ mice show significantly diminished OFF responses most likely caused by defective glycinergic neurotransmission via AII amacrine cells (Eulenburg et al., 2018). AII amacrine cells have been shown to be critical for the propagation of OFF-stimuli at least under scotopic conditions (Bloomfield and Dacheux, 2001), but there is also evidence that AII amacrine cells are also involved in the processing of cone-mediated photopic visual stimuli (Manookin et al., 2008). Thus, defective glycinergic signaling via AII amacrine cells is predicted to diminish OFF-signaling. This, however, is not seen in our behavioral analysis, suggesting that at least glycinergic neurotransmission involving AII amacrine cells does not contribute to this behavioral response. We cannot exclude, however, that a decreased inhibition caused by OFF-signals (Rabl et al., 2002) contributes to the observed increased reaction to ON preferring stimuli by a decreased crossover inhibition.

Consistent with the behavioral data, the ERG recordings confirmed that the overall signal processing within the retina of GlyT1^GlyT2-Cre^ mice is not severely affected, since only moderate changes were observed. These findings are consistent with previous studies demonstrating that the GlyR inhibitor strychnine has only moderate effects on ERGs recorded in rodents. It was reported previously that strychnine, a glycine receptor antagonist, caused a significant decrease in the amplitude of the OPs in the mouse retina (Liao et al., 2023). In GlyT1^GlyT2-Cre^ mice, however, significant OP reductions were not observed. A possible explanation for this discrepancy is that not all glycinergic amacrine cells are affected by GlyT2-Cre mediated GlyT1 gene inactivation (Eulenburg et al., 2018). Furthermore, GABAergic amacrine cells continue to support the generation of OPs and these might compensate at least partially the lack of glycinergic input.

Our detailed ERG recordings and analyses, including the assessment of the PhNR and the responses to sawtooth stimuli, indicate an asymmetry of the effects of retina specific GlyT1 deficiency on ON- and OFF-specific signal processing. These are consistent with previous findings showing distinct processing of ON- and OFF-signaling in the retina (Bowen et al., 1992), and extend these data by demonstrating differences in the involvement of glycinergic neurotransmission. Furthermore, these findings support the hypothesis that the defects in retinal processing observed in GlyT1^GlyT2-Cre^ mice are elicited within the inner retina. The observed heterogeneity of the effect of retinal GlyT1 deficiency on the PhNR suggests in addition to the AII amacrine cell at least one subtype of glycinergic amacrine cells is affected by the retinal GlyT1 gene inactivation in our GlyT1^GlyT2-Cre^ mouse line. Here, however, apparently only incomplete penetrance of GlyT1 inactivation is achieved. Which specific amacrine cell type is responsible for this effect, however, is unclear at present and requires further investigation. This, appears not to play a role for the observed behavioral effects, since a similar segregation of the behavior of GlyT1^GlyT2-Cre^ mice into discernable groups was not observed. Previous studies have shown, that at least in culture GlyT1 expression is also observed in Müller cells (Gadea et al., 1999), i.e., a retinal glial cell, which opens up the possibility that also this cell type contributes to changes in behavior and ERG induced by retinal GlyT1 deficiency. When analyzing GlyT1 expression in the retina, we found no expression in this cell type (Eulenburg et al., 2018) making this possibility highly unlikely.

As described above, previous studies have shown that in rodents, some of the effects described in this study might be mediated by glycine receptors of the GlyR α4 subtype (Jain et al., 2022). Since this subtype is predicted to be a pseudogene in humans, it is questionably if differences caused by GlyT1 deficiency are also detectable in humans. This question was addressed by our study of ERGs from a patient suffering from genetic induced GlyT1 deficiency. Here, no apparent structural defects were observed by imaging techniques in the patient retina and retinal function, as determined from ERG recording, indicated no apparent changes in scotopic function. In the photopic flash ERGs, however, the “photopic hill” phenomenon was missing. The amplitude decrease of the b-wave at high flash strengths that results in the establishment of the “photopic hill” in healthy humans has been attributed to the algebraic summation of the ON- and OFF-responses of the cone system during background illumination (Garon et al., 2014). Thus, the “photopic hill” phenomenon is believed to arise from the simultaneous responses of the ON-bipolar cells and of the OFF-bipolar cells to the instantaneous light stimulus (Hamilton et al., 2007; Garon et al., 2014). Here, the abolishment of the “photopic hill” phenomenon in the patient suffering from GlyT1 deficiency is consistent with a selective lowering of the contributions of OFF-bipolar cells to the ERG under background illumination. Unfortunately, a more extensive ERG analysis on the patient, also including a detailed assessment of ERG responses to ON/OFF sawtooth profiles was not possible, thus precluding a direct comparison of this aspect to our data obtained in GlyT1^GlyT2-cre^ mice.

This influence of GlyT1 deficiency on the light adapted flash ERG of humans can be associated with the prolonged b-wave kinetic in the photopic flash ERG in GlyT1^GlyT2-Cre^ mice. In rodents, however, no photopic hill effect can be observed, consistent with the findings that cones represent less than 3% of the photoreceptors in mice (Jeon et al., 1998) compared to about 5% cones in the human retina (Molday and Moritz, 2015). Therefore, a b-wave, that is predominantly carried by cone photoreceptors is nearly nonexistent (Gilmour et al., 2008).

In conclusion, our data demonstrate that GlyT1 deficiency result in significant changes in signaling processing in the inner retina of rodents and of a human patient, consistent with significant functional alteration of glycinergic amacrine cells within the inner retina. Although the core findings are different in mice and man, the underlying mechanism, i.e., the breakdown of glycinergic neurotransmission in the retina induced by GlyT1 deficiency is most likely the same. Whether the different defects detected in the human patient and in our mouse model for retinal GlyT1 deficiency are caused by dysfunction of the same glycinergic amacrine cell population, however, is at least questionable. Here, the precise mechanisms underlying the observed changes, however, remain unclear at present and deserve further investigation in future studies. The observed apparent differences between mouse and man underscores the importance for a detailed comparison of findings in different species to investigate the potential and validity of animal models for specific aspects of human diseases.

## Data Availability

The original contributions presented in the study are included in the article/supplementary material, further inquiries can be directed to the corresponding author.
